# Introduction to the History and Current Status of Evidence-Based Korean Medicine: A Unique Integrated System of Allopathic and Holistic Medicine

**DOI:** 10.1155/2014/740515

**Published:** 2014-04-14

**Authors:** Chang Shik Yin, Seong-Gyu Ko

**Affiliations:** ^1^Acupuncture and Meridian Science Research Center, Kyung Hee University, Seoul 130-701, Republic of Korea; ^2^Department of Preventive Medicine, Center for Clinical Research and Drug Development, Institution for Korean Medicine, Kyung Hee University, Seoul 130-701, Republic of Korea

## Abstract

*Objectives*. Korean medicine, an integrated allopathic and traditional medicine, has developed unique characteristics and has been active in contributing to evidence-based medicine. Recent developments in Korean medicine have not been as well disseminated as traditional Chinese medicine. This introduction to recent developments in Korean medicine will draw attention to, and facilitate, the advancement of evidence-based complementary alternative medicine (CAM). *Methods and Results*. The history of and recent developments in Korean medicine as evidence-based medicine are explored through discussions on the development of a national standard classification of diseases and study reports, ranging from basic research to newly developed clinical therapies. A national standard classification of diseases has been developed and revised serially into an integrated classification of Western allopathic and traditional holistic medicine disease entities. Standard disease classifications offer a starting point for the reliable gathering of evidence and provide a representative example of the unique status of evidence-based Korean medicine as an integration of Western allopathic medicine and traditional holistic medicine. *Conclusions*. Recent developments in evidence-based Korean medicine show a unique development in evidence-based medicine, adopting both Western allopathic and holistic traditional medicine. It is expected that Korean medicine will continue to be an important contributor to evidence-based medicine, encompassing conventional and complementary approaches.

## 1. Introduction

Korean medicine originates from prehistoric times and shares its origins with Chinese and Japanese medicine. However, compared with traditional Chinese medicine, Korean medicine is much less well-known. Although Korean and Chinese medicine have much in common, Korean medicine has developed on its own as a whole-person-centered medicine system [1], developing unique concepts and research, with “Four Constitution” (Sasang constitutional) medicine [2] and Saam acupuncture [3] being representative of genuinely Korean developments. In recent years, Korean medicine has become part of a national health system and its use has expanded [4]. Thus, by integrating traditional and modern approaches, Korean medicine constitutes an exemplary case of a national health system that encompasses many complementary and alternative approaches and has been active in validating and developing them.

What follows is a brief introduction to the characteristics of Korean medicine with a focus on evidence-based approaches, along with a historical review of a standard disease classification in Korean medicine as a representative example of the efforts being made to implement evidence-based medicine.

## 2. Korean Medicine: Tailored, Simple, and Practical

As indicated previously, the tradition of Korean medicine features an individualized approach based on constitutional energy traits of healthy qi [3], with simple and practical solutions, based on these underlying energy traits or core principles [5]. Qi and tao are the core principles that the body and the environment are composed of and by which both of them function [6]. Korean medicine places importance on self-discipline, a constitutional approach, and the three treasures (essence, qi, and spirit) deep in the axis of life [7] and focuses on the power and active response of the healthy qi as innate intelligence, intricately linked with constitutional characteristics and human dignity, which may be reflected and evaluated differentially in a pattern of systemic manifestations, including disease-specific ones that are directly linked to the pathology and nonspecific ones that may be remote from the lesion, with no apparent linkage to the pathology. These nonspecific characteristics may reflect the response of the body to the pathology rather than the pathology itself.

## 3. Challenges in Evidence-Based Approaches to CAM

Evidence-based medicine is about the best decision-making based on the best available evidence [8] and highlights a science and practice of medicine with a firm basis of evidence [9]. The evidence may vary in its hierarchical status and extent, but there may not be any strong opposition to the concept of a medicine being established and practiced based on stronger evidence. The evidence-based approach is rapidly gaining acceptance and expanding its realm, not only in academic [10, 11] or clinical activities but also with regard to regulation and insurance issues [12]. Complementary and integrative medicine is not an exception, even though there are many barriers, like the prioritization of classical books and practice as evidence sources [13].

In addition to the idea that traditional knowledge itself is an important source of evidence [14], the issue of evidence-based medicine is considered to be more challenging in the field of complementary and integrative medicine than in the field of the conventional allopathic medicine. Although a lack of research evidence for efficacy is said not to be evidence of a lack of efficacy [15], the main challenge is, of course, primarily due to the relative scarcity of evidence in the field of complementary and integrative medicine. However, in addition to the scarcity, it may be partially due to differences in the theoretical context and philosophical perspectives between complementary and integrative medicine and conventional allopathic medicine. This conceptual difference is accompanied naturally by differences in clinical practice. The theoretical context and clinical practice of complementary and integrative medicine have been handed down from the past or have developed from clinical experience rather than from the widely accepted current knowledge of modern biology, science, and technology, as is the case in orthodox allopathic medicine in which measurements are devised and performed on the basis of theoretical context, clinical practice, and technology and are linked directly with the production of high-quality scientific evidence [16]. Differences in the theoretical context, clinical practice, and measurements, in addition to the scarcity of evidence, complicate the issue of an evidence-based approach in the fields of complementary and integrative medicine.

## 4. Korean Medicine: An Exemplary Case in Evidence-Based Medicine

In the history of complementary and integrative medicine, Korea's situation may be a unique and exemplary case. Although many countries still lack national regulation of CAM, Korea established the parallel operation of two independent medical systems (Western medicine and Korean medicine) [17] in 1951, a rare instance whereby complementary and integrative approaches have been officially part of the national health system from the very start. In Korea, there is no generalist equivalent to a general physician in other countries. Each physician belongs to one of the two specialty professions: Western allopathic medicine or Korean medicine. Korean medicine is a specialty profession in which modern allopathic medicine and traditional, complementary approaches are integrated into a profession of modern holistic medicine. Only a few physicians satisfy the required educational and regulatory processes for both professions and receive both licenses from the government. Each is fully licensed to diagnose and treat patients and is independent in practicing medicine.

Korean medicine may be described as a form of integration of Western allopathic medicine and traditional medicine by considering the following points. (1) Educational programs for Korean medicine students and practitioners cover not only traditional knowledge but also the same basic and clinical medical sciences and research as in Western medicine. (2) Korean medicine practitioners are fully licensed to diagnose both disease entities of Western medicine origin and those of traditional medicine origin. (3) Treatment modalities provided by Korean medicine practitioners include those common in both Western and traditional medicine, as well as those specific to traditional medicine, such as acupuncture and yinyang balance concepts.

The Korean government has been promoting the evidence-based development of the national health system, especially with regard to Korean medicine, by means of standardization, research funding, and new drug development from herbal medicines. The Korean government has been expanding its research funding for such projects as Korean medicine diagnosis [18, 19] and new drug development from traditional herbal medicines [20, 21]. The Korean government invested a total of about 400 million US dollars during the first period to foster and develop Korean traditional medicine (2006–2010), with 63% invested in research and development projects, and planned to invest a total of about 1 billion US dollars during the 2nd period (2011–2015), with 34% invested in research and development projects [4].

As a fundamental platform for an evidence-based approach in modern Korean medicine, the history of a standard disease classification is briefly examined here. A standard disease classification alone does not mean, and cannot justify, the notion that Korean medicine is an evidence-based approach. However, a standard disease classification may be an indispensable fundamental basis for evidence-based medicine.

## 5. Standard Disease Classification in Korean Medicine

If any evidence of medical practice is collected, interpreted, and incorporated into evidence-based medicine, the clinical pictures of that medicine have to be captured, classified, and documented. A clinical picture that has not been captured and documented may not be used as evidence. If we are to capture the clinical picture of different systems of medicine, we may need different classification systems of the clinical entities targeted, evaluated, and treated with each system of medicine.

In an effort to capture the clinical picture and produce statistics on allopathic medicine and Korean medicine, different disease classification systems have been developed and stipulated as national standards in Korea. In the allopathic medicine field, a Korean adaptation of the World Health Organization (WHO) international classification of diseases (ICD) has been stipulated as the allopathic medicine volume for the Korean standard classification of diseases (KCD). The first edition of KCD appeared in 1952. It was revised in 1972, 1979, 1993, 2002, 2007, and 2010. In the Korean medicine field, the Korean medicine volume of KCD was first published in 1973 and was revised in 1979, 1995, and 2009. Recently, the KCD allopathic medicine volume and the KCD Korean medicine volume were integrated into one volume, “KCD6” in 2010 [22].

Although pattern identification for the overall analysis of symptoms and signs is a core component of the theoretical context and clinical practice of Korean medicine as well as traditional medicine in China [23] and Japan, the traditional way of pattern identification is still being developed as a standardized, validated diagnostic tool. A recent revision of the KCD Korean medicine volume (2009), which was then integrated into KCD6 in 2010, differed from previous revisions in several aspects. First, the hierarchy and appropriateness of the classification were thoroughly revised and improved. Second, the issue of possible overlap between allopathic medical entities and Korean medical entities was addressed in great detail and every effort was made to remove possible overlap. Possibly overlapping Korean medicine entities were replaced with existing KCD allopathic medicine entities. The remaining entities of genuine Korean medicine were classified under “U” codes. The genuine Korean medicine entities were classified into three categories: 97 diseases, 191 patterns/syndromes, and 18 diseases-patterns/syndromes from “Four Constitution” medicine. This may have been the first attempt in the history of disease classification in which a national standard successfully integrated allopathic medicine entities and genuine traditional medicine entities into one classification that is widely applicable to all major regulations, such as the national health insurance system, national health statistics, and the traffic accident insurance system.

In the Joseon dynasty, about four centuries ago, there was already a comprehensive disease classification, which was usually composed of descriptions on related anatomy, physiology, etiology, manifestations, pattern differentiation, self-discipline, qigong, and the simple herbs prescribed. However, the concepts of diseases, disorders, and patterns were not so clearly defined or differentiated [24]. Over the course of serial revisions to Korean medicine disease classifications in the 20th and 21st centuries, the concepts of diseases, disorders, and patterns have been differentiated more explicitly, with the differentiation concepts embodied in the classification structure and entities classified. In a recent revision, many traditional medicine codes were replaced with allopathic medicine codes, with the remaining classification of genuine traditional medicine diseases, disorders, and patterns fully and systematically integrated with the allopathic medicine classification.

Recently, WHO has also sought to integrate the International Classification of Traditional Medicine (ICTM) [25] into its standard classification of diseases (ICD) when revising ICD-10 to ICD-11 with the concept of ontology-based disease classification by 2015 [26]. KCD, the integrated classification of diseases encompassing allopathic medicine codes and genuine Korean medicine codes, was a valuable source for the WHO project. Indeed, it is an important issue in the ICD revision project that ICD categories are listed in a mutually exclusive and jointly exhaustive way to make them useful for such purposes as mortality statistics and morbidity statistics [27], which were also considered in the revision of KCD.

The standard classification of diseases will be an essential part of capturing the clinical pictures of medicine and a systematically organized classification will facilitate objective documentation, production of related statistics, and the contribution of complementary and integrative approaches to the general health of world citizens. In that sense, Korea's experiences in revising the national standard disease classification may well be an exemplary case to support and embody an evidence-based approach in institutionalizing complementary and integrative approaches and integrating them with conventional allopathic medicine.

## 6. Disorder and Pattern Coding in Korean Medicine

According to the coding guidelines for the WHO ICD-10, a main condition is defined as the condition primarily responsible for the patient's need for treatment or investigation. A main condition is diagnosed at the end of the episode of health care. If there is more than one condition that may be considered as a “main condition,” the one that was most responsible for the greatest use of resources is selected as a “main condition.” If no diagnosis was considered to be made, the main symptom or problem may be selected as a “main condition” [28].

Disease codes of Korean medicine are a combined form of Western medicine codes and traditional Korean medicine codes. Korean medicine codes are regulated by the law. According to the existing guideline for Korean medicine classifications [29], the followings are generally recommended.

In the first place, a “main condition” code is selected. Disease classification codes may be selected from the conventional Western medicine codes based on the conditions that patients appeal most or the amount of resources consumed in the clinical management. If the practicing physician does not consider the conventional Western medicine codes to be appropriate for the clinical picture of the patient, then the traditional Korean medicine codes, that is, U-codes in KCD, are used. When traditional Korean medicine codes in the U-codes are considered to be appropriate, the practicing physician should decide which category codes in the traditional Korean medicine codes are appropriate for the clinical picture of the patient: disorder codes, pattern codes, or “Four Constitution” medicine-related codes [29]. In addition to the “main condition,” other conditions that coexist or develop during the episode of health care and affect the management of the patient may also be listed [28]. In Korean medicine, codes for other conditions, as well as a code for a main condition, may be selected from both areas of Western medicine classification codes and traditional Korean medicine classification codes.

By following this practice, the practicing physician in Korean medicine may select a code that is considered to be most appropriate for the clinical picture of the patient, not only from the conventional Western medicine codes, but also from the traditional Korean medicine codes. Thus, selected disease coding may reflect the body of knowledge in Western medicine disease classification and traditional Korean medicine disease classification. In this sense, Korean medicine may be considered as an integration of conventional allopathic medicine and traditional holistic medicine.

A disease may be a set of dysfunctions in any of the body systems that may be defined by symptomatology, etiology, course and outcome, treatment response, linkage to genetic factors, and linkage to interacting environmental factors. A disorder/syndrome may be defined as a common pattern of similar symptoms in clinical practice. As to a disorder/syndrome, the etiology is not known or multiple etiologies are related in the clinical manifestations [27].

Disorders and patterns in traditional Korean medicine that are coded in the clinical practice of Korean medicine are the health care conditions that are responsible for the patient's need for treatment or investigation. Both the disorder and the pattern are diagnosed for the sake of treatment or investigation by practicing physician. Both are similar in that they are named after the body structures, causes, properties, severity, and so forth. However, the naming and concept of the disorder and the pattern in traditional Korean medicine usually deal with differential aspects of clinical pictures based on the theories of traditional medicine:

- A disorder in traditional medicine is a clinical picture that is relatively constant throughout the duration of that disorder. A pattern in traditional medicine is relatively temporary (constant/temporary).
- A disorder in traditional medicine usually delivers information reflecting the local manifestation of the pathology. A pattern in traditional medicine usually delivers information reflecting the systemic manifestation or the systemic response of the patient (local/systemic, pathology/patient).
- A disorder in traditional medicine is a concept that summarizes findings that are specific to the pathologic process under investigation. A pattern in traditional medicine means the pattern of combination of the manifestations that encompasses both specific symptoms/signs and nonspecific findings such as pulse diagnosis and tongue diagnosis (specific/nonspecific).
- A disorder may be applied for a time span. A disorder coding may be based on the main pathologic process which may show a causal relationship with the main manifestations in the patient. A pattern may be applied for a specific time span too. However, a pattern coding is based on the summarized whole picture that may be observed in the patient based on the perspectives of traditional medicine theories. A pattern is recognized based on the analysis of the systemic findings in the patient's body and mind which reflect the pathologic processes, responses to the pathologic processes, other concomitant findings, and innate or acquired constitutional traits of the patient (linear/multifactorial).
- A disorder in traditional medicine is usually described with general terms of anatomy-physiology together with terms of signs and symptoms. A pattern in traditional medicine is usually described in terms of the traditional medicine theories that are used to summarize the whole picture findings in the patients such as yin and yang balance, cold and heat, meridian, or constitution (general/theoretical).
- A disorder in traditional medicine is used to describe the general characteristics considered to be relatively common to general population. A pattern in traditional medicine is used to describe the individual characteristics considered to be relatively specific to the patient at that time (commonality/individuality).

The concept of a pattern may reflect constitutional characteristics of the patient in addition to the disease or disorder characteristics. In other words, the systemic active response and individual characteristics of the patient's body and mind may be the target of the concept of a pattern diagnosed in traditional Korean medicine. However, the disorder in traditional Korean medicine and the disorder or disease in conventional Western medicine primarily target the disordered or pathologic process itself disregarding the patient that is actively trying to recover from that disordered process and to maintain balanced state of health. Considering the abovementioned concepts, the pattern and the disorder-disease may be considered as two complementary aspects that may be targeted when trying to capture the clinical picture of health care conditions.

## 7. Conclusions

Korean medicine has been an active player, at the forefront, in the implementation of evidence-based medicine. Korean medicine, an integrated conventional allopathic medicine and traditional holistic medicine, is exemplary in developing complementary and integrative approaches into an essential part of evidence-based mainstream medicine, implementing a national standard disease classification encompassing both allopathic and genuine traditional concepts. In this paper, recent developments regarding the biological activities of herbal medicines, diagnostic evaluations, and clinical applications are introduced. Korean medicine is expected to be an important contributor to the establishment and implementation of evidence-based, tailored medicine, integrating complementary and conventional approaches.
